# Associations between antagonistic SNPs for neuropsychiatric disorders and human brain structure

**DOI:** 10.1038/s41398-024-03098-1

**Published:** 2024-10-02

**Authors:** Lydia M. Federmann, Friederike S. David, Christiane Jockwitz, Thomas W. Mühleisen, Dominique I. Pelzer, Markus M. Nöthen, Svenja Caspers, Katrin Amunts, Janik Goltermann, Till F. M. Andlauer, Frederike Stein, Katharina Brosch, Tilo Kircher, Sven Cichon, Udo Dannlowski, Lisa Sindermann, Andreas J. Forstner

**Affiliations:** 1 Institute of Neuroscience and Medicine (INM-1), Research Centre Jülich, Jülich, Germany; 2 Institute of Human Genetics, University of Bonn, School of Medicine & University Hospital Bonn, Bonn, Germany; 3 Institute for Anatomy I, Medical Faculty & University Hospital Düsseldorf, Heinrich Heine University, Düsseldorf, Germany; 4 Cécile and Oskar Vogt Institute for Brain Research, Medical Faculty & University Hospital Düsseldorf, Heinrich Heine University, Düsseldorf, Germany; 5 Department of Biomedicine, University of Basel, Basel, Switzerland; 6 Institute for Translational Psychiatry, University of Münster, Münster, Germany; 7 Department of Neurology, Klinikum rechts der Isar, School of Medicine, Technical University of Munich, Munich, Germany; 8 Department of Psychiatry and Psychotherapy, Philipps-University and University Hospital Marburg, Marburg, Germany; 9 Institute of Behavioral Science, Feinstein Institutes for Medical Research, Manhasset, New York, USA; 10 Institute of Medical Genetics and Pathology, University Hospital Basel, Basel, Switzerland; 11 Centre for Human Genetics, Philipps-University Marburg, Marburg, Germany

**Keywords:** Molecular neuroscience, Psychiatric disorders

## Abstract

A previously published genome-wide association study (GWAS) meta-analysis across eight neuropsychiatric disorders identified antagonistic single-nucleotide polymorphisms (SNPs) at eleven genomic loci where the same allele was protective against one neuropsychiatric disorder and increased the risk for another. Until now, these antagonistic SNPs have not been further investigated regarding their link to brain structural phenotypes. Here, we explored their associations with cortical surface area and cortical thickness (in 34 brain regions and one global measure each) as well as the volumes of eight subcortical structures using summary statistics of large-scale GWAS of brain structural phenotypes. We assessed if significantly associated brain structural phenotypes were previously reported to be associated with major neuropsychiatric disorders in large-scale case-control imaging studies by the ENIGMA consortium. We further characterized the effects of the antagonistic SNPs on gene expression in brain tissue and their association with additional cognitive and behavioral phenotypes, and performed an exploratory voxel-based whole-brain analysis in the FOR2107 study (*n* = 754 patients with major depressive disorder and *n* = 847 controls). We found that eight antagonistic SNPs were significantly associated with brain structural phenotypes in regions such as anterior parts of the cingulate cortex, the insula, and the superior temporal gyrus. Case-control differences in implicated brain structural phenotypes have previously been reported for bipolar disorder, major depressive disorder, and schizophrenia. In addition, antagonistic SNPs were associated with gene expression changes in brain tissue and linked to several cognitive-behavioral traits. In our exploratory whole-brain analysis, we observed significant associations of gray matter volume in the left superior temporal pole and left superior parietal region with the variants rs301805 and rs1933802, respectively. Our results suggest that multiple antagonistic SNPs for neuropsychiatric disorders are linked to brain structural phenotypes. However, to further elucidate these findings, future case-control genomic imaging studies are required.

## Introduction

Neuropsychiatric disorders − such as attention deficit hyperactivity disorder (ADHD), anorexia nervosa (ANO), autism spectrum disorder (ASD), bipolar disorder (BIP), major depressive disorder (MDD), obsessive-compulsive disorder (OCD), schizophrenia (SCZ), and Tourette’s syndrome (TS) − are complex and common brain disorders with lifetime prevalences of 0.7 to 16.6% [1–5]. These neuropsychiatric disorders tend to co-occur and partially share core symptoms like negative affect and cognitive deficits [6] suggesting the presence of similarities at the neurobiological level [7, 8].

Large-scale genomic and imaging datasets have enabled insights into the genetic architecture and the neuroimaging correlates of neuropsychiatric disorders [9–11]. The large-scale magnetic resonance imaging (MRI) case-control studies by the Enhancing NeuroImaging Genetics through Meta-Analysis (ENIGMA) consortium uncovered robust findings in brain structural alterations in patients with neuropsychiatric disorders compared to controls [10]. Similarities in brain structural alterations were observed across disorders, especially in the mood and psychosis spectrum, in addition to disorder-specific brain structural alterations [12, 13]. Moreover, the similarity of neuroimaging profiles tends to coincide the genetic correlations between neuropsychiatric disorders reported in previous studies (see below), indicating that similarities of case-control differences are at least partly accounted for by shared genetic risk [14, 15].

Genome-wide association studies (GWAS) identified hundreds of genetic loci associated with neuropsychiatric disorders [16] and discovered a substantial genetic overlap among these disorders [17–19]. Beyond that, studies on genetic differences between diagnostic categories revealed SNPs specific to one neuropsychiatric disorder [20–22]. In addition, antagonistic effects, i.e. genetic factors increasing the risk for one neuropsychiatric disorder while being protective for another disorder, were observed across various levels from alleles, genes, to tissue-specific gene expression [23]. In particular, antagonistic single-nucleotide polymorphisms (SNPs) at eleven genomic loci were identified in the second cross-disorder GWAS meta-analysis of the Psychiatric Genomics Consortium (PGC-CDG2) [24] that comprised 232 964 cases across eight neuropsychiatric disorders (ADHD, ANO, ASD, BIP, MDD, OCD, SCZ, and TS). The antagonistic SNPs at the eleven loci showed *p* ≤ 1 × 10^−6^ in the cross-disorder meta-analysis and presented effects with opposite directions for at least two disorders [24]. Information on the antagonistic SNPs including their associations with the individual neuropsychiatric disorders can be found in the Supplementary Table S3.3 of the PGC-CDG2 GWAS meta-analysis [24].

Antagonistic SNPs might be of particular interest for understanding neuropsychiatric disorders as these variants may characterize functional mechanisms that influence opposed manifestations in specific phenotypical dimensions despite the known genetic and phenotypic overlaps between neuropsychiatric disorders. For example, ASD and SCZ are both characterized by social and cognitive difficulties [25, 26]. In relation to the ability of mentalizing, however, patients with these disorders may represent opposite extremes as patients with ASD were reported to present deficits in attributing intentions of agency, while patients with SCZ showed increased attribution of intentions [27].

Although functional mapping and annotation of GWAS results were greatly facilitated by platforms like FUMA [28], functional characterization of the top-associated antagonistic SNPs at the eleven loci was not conducted in the PGC-CDG2 GWAS meta-analysis [24]. Such an analysis, however, is relevant for understanding the biological consequences of these SNPs, such as, for example, the influence of these SNPs on brain region-specific gene expression (expression quantitative trait locus (eQTL)) [29, 30].

The annotation of SNPs for neuropsychiatric disorders has become particularly important in regard to brain structure which is considered as a central intermediate phenotype for neuropsychiatric disorders as genetic factors might mediate the disease risk via changes at the brain structural level [31, 32]. Previous studies reported shared genetic variants [33, 34] and significant genetic correlations between brain structural phenotypes and neuropsychiatric disorders [35–38]. Furthermore, polygenic risk scores for neuropsychiatric disorders were shown to be significantly associated with brain structure (e.g. [39]). These genetic links between neuropsychiatric disorders and brain structural phenotypes have great potential to pinpoint the underlying neurobiological processes of disease susceptibility [40]. For the top-associated antagonistic SNPs at the eleven loci of the PGC-CDG2 study [24], however, no systematic investigation on their association with brain structural phenotypes has yet been reported.

Against this backdrop, our overall aim in the present study was to further characterize the pathophysiological mechanisms of the eleven antagonistic SNPs of neuropsychiatric disorders identified in the PGC-CDG2 GWAS meta-analysis [24]. Having underlined the importance of studying brain structure, we first investigated the association between the eleven antagonistic SNPs and brain structural phenotypes. Herein, we hypothesized that the influence of an antagonistic SNP on brain structure might lead to its protective effect for one disorder and increased risk for another disorder. Second, we assessed if the significantly associated brain structural phenotypes had already been described to be altered in patients with neuropsychiatric disorders compared to controls. In this context, we presumed that the antagonistic SNPs are associated with the structure of brain regions known to be implicated in multiple neuropsychiatric disorders. Third, we aimed to further characterize the antagonistic SNPs by examining their links to gene expression in the brain and their association with further traits. Fourth, we performed an exploratory voxel-wise whole-brain analysis in the FOR2107 cohort to identify potential brain structure associations of antagonistic SNPs at the voxel-wise level. Thereby, we extended the analysis beyond atlas-derived brain structural phenotypes.

## Materials and Methods

**Fig. 1:**
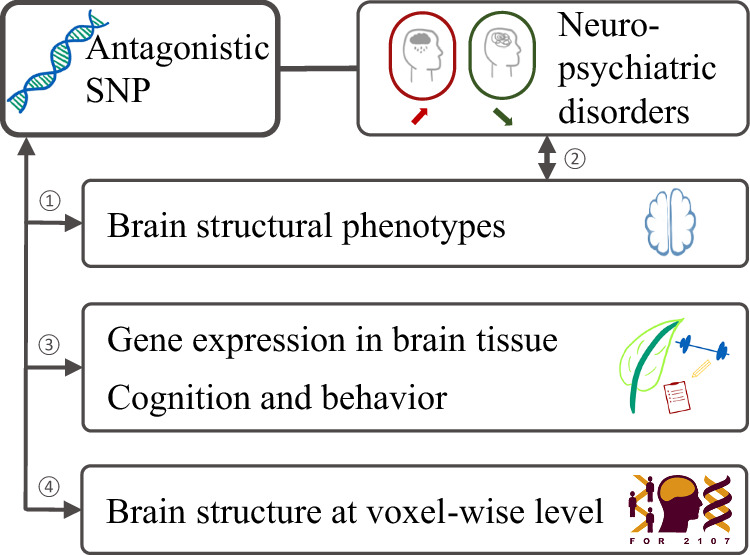
Schematic overview of the four-folded approach. Our analyses systematically characterize the eleven antagonistic SNPs with regard to their link to brain structure and brain-related traits: In (1) we perform a SNP to brain image-derived phenotype (IDP) analysis. In (2) we investigate if implicated IDPs are altered in patients with neuropsychiatric disorders compared to controls using the ENIGMA datasets. In (3) we assess if antagonistic SNPs are part of eQTLs for brain tissue, and if antagonistic SNPs are associated with additional cognitive and behavioral traits. In (4) we investigate if there are further associations of the antagonistic SNPs with brain structure at the voxel-wise level that might have been missed in the SNP-to-IDP analysis. SNP single-nucleotide polymorphism.

We characterized the top SNPs at eleven antagonistic loci for neuropsychiatric disorders at the brain level using a four-folded approach (Fig. 1). The study was approved by the local ethics committees of the University of Marburg (AZ: 07/14) and the University of Münster (AZ: 2014-422-b-S), Germany. All methods presented in this manuscript were conducted in accordance with relevant guidelines and regulations. Informed consent was obtained from all participants.

### Association Between Antagonistic SNPs and Brain Structure

First, we investigated the association between the eleven antagonistic SNPs [24] and brain image-derived phenotypes (IDPs) by examining summary statistics of large-scale GWAS from the ENIGMA consortium [35, 38] and the ENIGMA-CHARGE collaboration [41]. Thereby, we report the associations between the eleven antagonistic SNPs [24] and 78 IDPs using summary statistics from GWAS of cortical thickness (CT) and surface area (SA) [35], GWAS of hippocampal volume [41], and GWAS of subcortical volumes [38] (Supplementary Table S1). The respective GWAS comprised *n* = 33 281 (CT and SA; [35]), *n* = 26 814 (hippocampal volume; [41]), and *n* = 37 741 (subcortical volumes; [38]) individuals in the discovery cohort, providing sufficient power to detect genetic signals with small effect sizes. The studies were approved by the respective ethics committees and informed consent was obtained for all participants as described in the respective studies [35, 38, 41]. The 78 IDPs comprised CT and SA for 34 regions of interests (ROIs, averaged across both hemispheres, [35]) each as delineated by the Desikan-Killiany (DK) atlas [42], overall SA, average CT, and the volume of the following subcortical structures: amygdala, nucleus accumbens, brainstem, caudate, globus pallidus, hippocampus, putamen, and thalamus. Subcortical structures were segmented by each participating site from MRI using FreeSurfer or FSL-FIRST [38, 43, 44].

Statistics of the SNP-to-IDP association (e.g. *p*-value, effect size, and effect allele) were queried using the ENIGMA-Vis tool [45]. The most recent GWAS of subcortical volumes by Satizabal et al. [38] was not covered in ENIGMA-Vis and thus, the SNP-to-IDP associations for these phenotypes were extracted from the corresponding GWAS summary statistics provided by the authors of that study. We corrected for multiple testing with the Benjamini-Hochberg (BH) false discovery rate (FDR) procedure [46] and considered associations with *p*_FDR_ < 0.05 as significant. We note that the SNP rs1933802 was not included in the GWAS summary statistics of Grasby et al. [35] and was thus substituted by the proxy SNP rs314280 for the SNP-to-IDP analysis (*r*^2^ = 1) based on linkage disequilibrium (LD) in Utah residents with Northern and Western European ancestry (CEU) using the LDproxy tool [47].

In addition to this SNP-to-IDP analysis, we performed bootstrapping to test whether the number of significant SNP-to-IDP associations differed for the eleven antagonistic SNPs in comparison to randomly sampled sets of eleven SNPs (see Supplementary Information Note 1 for details).

### Alterations of implicated brain structures in patients with neuropsychiatric disorders

Second, we explored if IDPs that were significantly associated with an antagonistic SNP have previously been reported to be altered in patients with neuropsychiatric disorders based on ENIGMA datasets. In particular, we were interested if the reported brain structural alterations had opposite directions between the two disorders implicated by the antagonistic SNP. To contrast SNP associations and brain structural alterations, we compared the effect size directions of (i) SNP-to-IDP associations, (ii) SNP to disease risk, and (iii) alterations of the IDP observed in patients with neuropsychiatric disorders compared to controls. This analysis was based on the summary statistics of case-control MRI studies by the ENIGMA consortium (see Supplementary Table S2 for an overview of the included ENIGMA studies and their sample sizes and cohort characteristics). The studies were approved by the respective ethics committees, and each study obtained informed consent for the participants of all cohorts (cf. descriptions in the original studies). We used the ENIGMA Toolbox [48] to retrieve the summary statistics. These included *p*-values corrected for multiple testing, which we considered significant at *p*_adjusted_ < 0.05. Here, we note that different multiple testing correction procedures were applied for subcortical and cortical IDPs (see Supplementary Table S2 for further details). Furthermore, we excluded disorder-specific subphenotypes (e.g. recurrent episodes of depression, or bipolar subtype) and did not assess case-control differences for ANO and TS patients since, at the time of analysis, no large-scale imaging study for ANO and TS has been published.

### Gene expression in the brain

Third, to further characterize the antagonistic SNPs we conducted follow-up analyses by (i) reviewing whether the antagonistic SNPs are part of eQTLs for different brain tissues, and (ii) reporting further trait associations beyond neuropsychiatric disorders (see below). To identify the link between the antagonistic SNPs and gene expression levels, we queried eQTL data in twelve brain tissues of the Genotype-Tissue Expression database (GTEx v8) [49] (see Supplementary Table S3 for an overview of brain tissues) and in brain tissues of the frontal, occipital, and temporal cortex as well as in the average across all the brain tissues of the Brain eQTL Almanac (BRAINEAC) database [50]. We reported antagonistic SNPs as a significant part of an eQTL using a threshold of *p* < 4.0 × 10^−04^, corresponding to a Bonferroni correction for multiple testing for eight SNPs and 16 brain tissues. eQTLs of pseudogenes were excluded according to the ‘locus type’ reported in the HUGO Gene Nomenclature Committee (HGNC) database (https://www.genenames.org) [51]. We replaced rs75595651 by the proxy SNP rs77087420 (*r*^2^ = 1 in CEU) using the LDproxy tool [47] as rs75595651 was not present in the eQTL databases.

### Further trait associations

To examine the link between the antagonistic SNPs and further traits relevant to cognitive and behavioral processes (e.g., education, chronotype, food preferences, and neuroticism), we retrieved associations from Open Targets Genetics [52, 53] with *p* < 5.0 × 10^−08^. The Open Targets Genetics portal maintains trait associated loci from the NHGRI-EBI GWAS Catalog [54], and published GWAS analyses using data of the UK Biobank (cf. [55] and https://www.nealelab.is/uk-biobank/*)*. We excluded trait associations with any of the eight neuropsychiatric disorders included in the PGC-CDG2 GWAS meta-analysis [24].

### Voxel-wise whole-brain analysis in the FOR2107 study

Fourth, we investigated voxel-wise gray matter volume (GMV) differences related to the allelic status in a subsample of the FOR2107 study [7, 56]. The FOR2107 is an ongoing bi-center study that recruits healthy controls (HC) and patients along the affective disorders-psychosis spectrum in Marburg and Münster, Germany. In detail, we performed the voxel-wise whole-brain analysis in *n* = 847 HC and *n* = 754 patients with MDD of European ancestry that passed genetic and MRI quality control. This sub-sample of participants included 64.2% females and presented a mean age of 35.4 years (SD 13.1 years). All participants provided written informed consent, and ethical approval was obtained from the local ethics committees in Marburg and Münster, Germany. Further information on the study characteristics, MRI acquisition, preprocessing, and the genomic data of the FOR2107 study can be found in the Supplementary Information (Note 2) as well as in previous publications [7, 56–58].

We tested the influence of the eleven antagonistic SNPs on voxel-wise GMV using the CAT-12 toolbox (version 2159) [59] which builds on the SPM12 toolbox (version 7771) [60]. We used general linear models to assess positive and negative associations between the genotype dosage of one SNP and GMV while age, sex, diagnosis, total intracranial volume, scanner body coil (differing for some study participants in Marburg, Germany), and the first three components of a multidimensional scaling analysis to control for population stratification were included as covariates. Associations of clusters passing an initial cluster-forming threshold of *p*_uncorrected_ < 0.001 with an extended threshold of cluster size *k >* 10 were reported and annotated using the automated anatomical labeling atlas version 3 (AAL) [61, 62]. We applied the peak-level family wise error (FWE) correction for multiple testing and considered results significant at *p*_FWE_ < 0.05. To provide a more fine-grained mapping, peak voxels of GMV clusters that were significantly associated with allele dosage at *p*_FWE_ < 0.05 were further annotated using the cytoarchitectonic maps of the Julich Brain Atlas version 3.1 [63].

## Results

### Association between antagonistic SNPs and brain structure

**Fig. 2:**
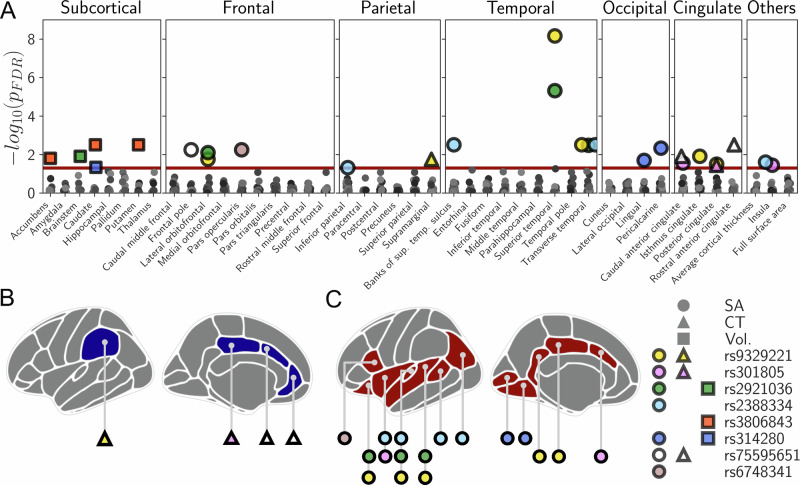
Significant associations between the antagonistic SNPs and IDPs. **A** presents the SNP-to-IDP associations (*p*_FDR_ < 0.05, red line) color-coded by SNP. Note that we replaced rs1933802 using the proxy rs314280 as described in the Materials and Methods. Brainplots present significant SNP-to-IDP associations for CT (**B**) and SA (**C**). CT cortical thickness, FDR false discovery rate, IDP image-derived phenotype, SA surface area, SNP single-nucleotide polymorphism, sup superior, temp temporal, Vol Volume.

Eight of the eleven examined antagonistic SNPs were significantly associated with at least one IDP after correction for multiple testing. The IDPs included 13 SA and four CT measurements, as well as five subcortical volumes (Fig. 2, Table 1). Implicated brain regions were widespread across the entire cortex. In particular, rs9329221 and rs2921036, two SNPs with antagonistic effects on ASD vs. SCZ, showed the strongest association with an IDP, namely the SA of the superior temporal region (rs9329221: *p*_FDR_ = 6.9 × 10^−09^; rs2921036: *p*_FDR_ = 4.8 × 10^−06^). Using a bootstrapping test, we showed that the number of significant SNP-to-IDP associations for the antagonistic SNPs was significantly higher than for eleven random non-antagonistic SNPs from the same GWAS summary statistics (*p =* 1.0 × 10^−04^) or for SNPs with cross-disorder associations (*p* ≤ 1.0 × 10^−06^) in the PGC-CDG2 GWAS meta-analysis (*p =* 3.0 × 10^−03^) (for more details see Supplementary Information Note 1).

**Table 1:** Statistics of the significant associations between eight antagonistic SNPs and IDPs.

rsID	CHR	BP	EA/OA	Risk	Prot.	IDP	CT/SA/Vol.	*p*-value	*p*_FDR_-value	Effect
rs2388334	6	98591622	A/G	TS	ASD	Transverse temporal	SA	2.16 × 10^−05^	3.13 × 10^−03^	1.643
					BIP	Banks of sts.	SA	2.89 × 10^−05^	3.14 × 10^−03^	−3.548
						Insula	SA	6.04 × 10^−04^	2.50 × 10^−02^	4.445
						Inferior parietal	SA	1.50 × 10^−03^	4.83 × 10^−02^	−10.312
rs301805	1	8481016	T/G	MDD	SCZ	Caudal ant. cingulate	SA	7.09 × 10^−04^	2.80 × 10^−02^	−2.571
						Insula	SA	1.00 × 10^−03^	3.62 × 10^−02^	4.277
						Posterior cingulate	CT	1.05 × 10^−03^	3.65 × 10^−02^	0.003
rs75595651	4	123133540	T/C	BIP	MDD	Rostral ant. cingulate	CT	2.87 × 10^−05^	3.14 × 10^−03^	0.013
						Frontal pole	SA	7.88 × 10^−05^	5.71 × 10^−03^	2.018
						Caudal ant. cingulate	CT	2.16 × 10^−04^	1.24 × 10^−02^	0.013
rs314280^1^	6	105365891	A/G	SCZ	MDD	Pericalcarine	SA	5.39 × 10^−05^	4.68 × 10^−03^	−6.217
						Lingual	SA	4.68 × 10^−04^	2.03 × 10^−02^	−8.131
						Caudate	Vol.	1.39 × 10^−03^	4.64 × 10^−02^	3.197
rs6748341	2	225377574	C/G	SCZ	ANO	Pars opercularis	SA	7.55 × 10^−05^	5.71 × 10^−03^	5.788
rs3806843	5	140212538	T/C	SCZ	MDD	Putamen	Vol.	1.64 × 10^−05^	3.13 × 10^−03^	−4.310
						Caudate	Vol.	2.10 × 10^−05^	3.13 × 10^−03^	−4.254
						Nucleus accumbens	Vol.	3.10 × 10^−04^	1.58 × 10^−02^	−3.607
rs9329221	8	10240202	T/G	SCZ	ASD	Superior temporal	SA	7.93 × 10^−12^	6.89 × 10^−09^	−12.496
						Transverse temporal	SA	1.33 × 10^−05^	3.13 × 10^−03^	−1.707
						Isthmus cingulate	SA	2.04 × 10^−04^	1.24 × 10^−02^	3.264
						Supramarginal	CT	3.84 × 10^−04^	1.77 × 10^−02^	0.002
						Lateral orbitofrontal	SA	3.87 × 10^−04^	1.77 × 10^−02^	5.045
						Posterior cingulate	SA	8.46 × 10^−04^	3.19 × 10^−02^	−2.924
rs2921036	8	8363897	T/C	ASD	SCZ	Superior temporal	SA	1.11 × 10^−08^	4.82 × 10^−06^	10.374
						Transverse temporal	SA	3.46 × 10^−05^	3.34 × 10^−03^	1.615
						Lateral orbitofrontal	SA	1.20 × 10^−04^	8.02 × 10^−03^	−5.439
						Brainstem	Vol.	2.28 × 10^−04^	1.24 × 10^−02^	3.685

EA refers to the effect allele of disorder risk (increased risk or protective effect) [24] as well as the effect allele in relation to the *p*-value and effect size, which were taken from summary statistics of the respective GWAS of brain phenotypes [35, 38, 41]. The effect is given as *β* for cortical IDPs and as *Z*-scores for subcortical IDPs. ^1^Note that we replaced rs1933802 using the proxy rs314280 as described in the Materials and Methods.

*ANO* anorexia nervosa, *ant.* anterior, *ASD* autism spectrum disorder, *BIP* bipolar disorder, *CT* cortical thickness, *EA* effect allele, *FDR* false discovery rate, *IDP* image-derived phenotype, *MDD* major depressive disorder, *OA* other allele, *Prot.* protective, *SA* surface area, *SCZ* schizophrenia, *SNP* single-nucleotide polymorphism, *sts.* superior temporal sulcus.

### Alterations of implicated brain structures in patients with neuropsychiatric disorders

**Fig. 3:**
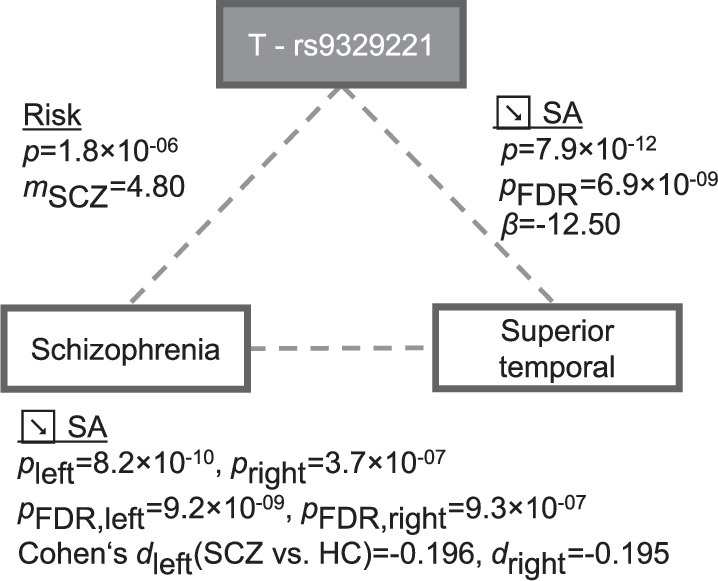
Association of the SNP rs9329221 with SCZ and SA measures of the superior temporal region. The T allele of the SNP rs9329221 was linked to SCZ risk [24] and was associated with a decrease of SA within the Desikan-Killiany region superior temporal [35]. This region showed prominent decrease of SA in patients with SCZ compared to controls (Table S5a in [64]). We note that Fig. 3 displays association results of the different individual investigations and does not represent a separate mediation analysis. FDR false discovery rate, HC healthy control, SA surface area, SCZ schizophrenia, SNP single-nucleotide polymorphism.

None of the significantly associated IDPs had previously been reported to be positively associated with one neuropsychiatric disorder and - at the same time - negatively associated with another. However, we observed significant case-control differences for CT measurements of the associated IDPs for BIP, MDD, and SCZ (Supplementary Table S4). Case-control differences for SA measurements of the associated IDPs were merely observed for SCZ. For example, patients with SCZ compared to controls showed a decrease in SA in the region of the superior temporal gyrus (*p*_FDR, left_ = 9.2 × 10^−09^, Cohen’s d_left_ (SCZ vs. HC)=−0.196; *p*_FDR, right_ = 9.3 × 10^−07^, *d*_right_ = −0.195) [64]. Notably, the T allele of rs9329221, associated in the present study with decreased SA (*p*_FDR_ = 6.9 × 10^−09^, *β* *= −*12.50), also increases SCZ risk (Fig. 3).

### Gene expression in the brain

Six of the eight antagonistic SNPs that were significantly associated with at least one IDP were part of eQTLs in brain tissue (Supplementary Table S3). The statistically most robust associations were observed for rs2921036 and rs3806843: The C allele of rs2921036 (increased risk for SCZ and protective against ASD) was linked to reduced expression of the long non-coding RNA *FAM85B* in the entire cortex (normalized effect size (NES) in GTEx: NES = −0.67, *p =* 3.0 × 10^−16^), the cerebellum (NES = −0.69, *p =* 1.4 × 10^−15^), and the nucleus accumbens (NES = −0.63, *p =* 3.6 × 10^−14^) among other brain tissues. Furthermore, rs3806843, an intronic variant within the Protocadherin Alpha (*PCDHA*) cluster, was part of an eQTL regulating the expression of several members of this gene family. The C allele of rs3806843 (increased risk for MDD and protective against SCZ) upregulated the expression of *PCDHA1* in the cerebellum (NES = 0.56, *p =* 4.0 × 10^−15^) as well as the expression of *PCDHA13* in the cerebellar hemisphere (NES = 0.55, *p =* 2.9 × 10^−14^) and the entire cortex (NES = 0.53, *p =* 2.2 × 10^−12^).

### Further trait associations

Annotation of antagonistic SNPs revealed associations to cognitive and behavioral traits for all eight SNPs (Supplementary Table S5) with the strongest associations being found for rs2921036 and rs2388334: The C allele of rs2921036 (higher risk for SCZ and protective against ASD) was associated with lower measurements of neuroticism (*p =* 6*.*2 × 10^−26^; https://www.nealelab.is/uk-biobank/). The G of rs2388334 (increased risk for BIP and ASD and protective against TS) was linked to higher college or university degree (*p =* 2.8 × 10^−37^; https://www.nealelab.is/uk-biobank/*)*, higher measures of intelligence (*p =* 3.7 × 10^−29^; [65]), and higher cognitive performance (*p =* 1.8 × 10^−26^; [66]).

### Voxel-wise whole-brain analysis in the FOR2107 study

**Fig. 4:**
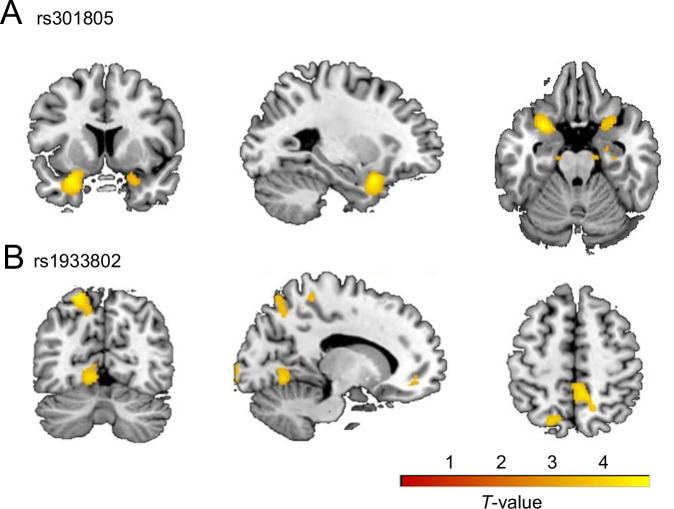
Associations of rs301805 and rs1933802 with gray matter volume in the FOR2107 study. Associations of the G allele dosage of rs301805 (**A**) and the G allele dosage of rs1933802 (**B**) with GMV with *p*_uncorrected_ < 0.001 and *k* > 10. The peak voxels and anatomical labels of the GMV clusters are provided in Table S6. Furthermore, associations of rs301805 and rs1933802 with GMV that remained significant at *p*_FWE_ < 0.05 are shown in Figure S2. FWE family-wise error, GMV gray matter volumes.

All eleven antagonistic SNPs showed association between GMV and allele dosage in data of the FOR2107 study with *p*_uncorrected_ < 0.001 (Supplementary Table S6). After correction for multiple testing, we observed a significant negative association of the G allele dosage of rs301805 and GMV in the left superior temporal pole (*k* = 998, *x*/*y*/*z* = −28/10/−22, *T* = 4.85, *p*_uncorrected_ = 6.7 × 10^−7^; *k* = 44, *p*_FWE_ = 1.2 × 10^−2^). Based on the Julich Brain Atlas v3.1 the peak voxel was assigned to the Frontal-to-Temporal-II GapMap [63] (Figure S2A). Furthermore, we found a significant positive association of the G allele dosage of rs1933802 and GMV in the left superior parietal region (*k* = 448, *x*/*y*/*z* = −20/−69/62, *T* = 4.62, *p*_uncorrected_ = 2.1 × 10^−6^; *k* = 15, *p*_FWE_ = 2.9 × 10^−2^). The peak voxel of this cluster was mapped to the Area 7A of the superior parietal lobe based on the Julich Brain Atlas v3.1 [63] (Fig. S2B). The results of both SNPs are visualized in Fig. 4 (brainplots showing GMV clusters with *p*_uncorrected_ < 0.001). For the other nine SNPs, no significant results were found after correction for multiple testing (*p*_FWE_ > 0.05).

## Discussion

In the present study, we further characterized eleven SNPs from antagonistic loci identified by the PGC-CDG2 GWAS meta-analysis [24] with a special focus on their link to brain structure. Herein, we presumed that a SNP’s antagonistic effect on two neuropsychiatric disorders might be shaped by its influence on brain structure. In our four-folded approach, we firstly showed that eight antagonistic SNPs with opposite directional effects on neuropsychiatric disorders were associated with at least one brain structural phenotype (SNP-to-IDP analysis). Secondly, we found that, while no opposite directions of effect were observed between disorders, case-control differences in CT and SA measurements of the associated IDPs were present for BIP, MDD, and SCZ. Thirdly, we reported that antagonistic SNPs affected gene expression in the implicated brain regions as well as behavioral and cognitive traits. Lastly, the voxel-wise whole-brain analysis in the FOR2107 study revealed significant associations between GMV and two antagonistic SNPs (rs301805 and rs1933802).

In our SNP-to-IDP analysis, we showed that the eleven antagonistic SNPs were associated with a higher number of IDPs when compared to the sampling distribution of randomly sampled sets of eleven SNPs. It is thus possible that antagonistic SNPs might mediate their risk for developing a specific neuropsychiatric disorder via changes at the brain structural level.

Several associated regions - such as the anterior cingulate cortex and the superior temporal gyrus - were previously reported in relation to symptoms and structural changes observed in patients with neuropsychiatric disorders [12, 67, 68]. For example, the T allele of the antagonistic SNP rs75595651 (increased risk for BIP and protective against MDD) was associated with increased CT in the caudal and rostral anterior cingulate cortex. This region plays an important role for emotional processing [69] and was suggested to be thinner in patients with MDD compared to controls [70]. Other examples are rs2921036 and rs9329221 (ASD vs. SCZ; LD between both SNPs in CEU: *r*^2^ = 0.46) that were associated with the SA of the superior temporal gyrus. This region encloses areas relevant for social cognition and language processing [71–74], which tend to be altered in patients of ASD [72] and SCZ [75–77]. Furthermore, in the context of mentalizing tasks, patients with ASD and SCZ even showed opposed neural correlates related to the connectivity of posterior parts of the superior temporal sulcus [27].

We did not uncover structural alterations with opposite directions in any of the implicated IDPs using the case-control MRI studies of neuropsychiatric disorders by the ENIGMA consortium, meaning IDPs were not found to be increased in patients of one disorder compared to controls and at the same time decreased in patients of another disorder. As variation in brain structure is influenced by many common genetic variants with small effect sizes [35] as well as other genetic and environmental factors, the effects of individual SNPs might not have been apparent in brain structural alterations at the case-control analysis level [58]. Furthermore, brain structural alterations in patients were reported to be overlapping across neuropsychiatric disorders [12–14, 68] and these similarities tend to follow cross-disorder genetic correlations [15] as well as shared molecular features [78]. Future studies are required to point out structural alterations that differ or are even opposed among two patient groups and relate these to genetic differences across neuropsychiatric disorders.

However, our findings might give a first notion on how an antagonistic SNP could be linked to brain structure. In particular, for the SNP rs9329221 (T allele increased SCZ risk and was protective against ASD), we found that the T allele was linked to decreased SA in the superior temporal region that in turn was previously found to be decreased in patients with SCZ [64]. Although no alterations of SA in patients with ASD have been observed in the respective ENIGMA case-control MRI study [79], the GMV in the right superior temporal gyrus was shown to be increased in healthy children with autistic traits [80]. Moreover, opposed GMVs (increased in patients with ASD and decreased in patients with SCZ) were observed within middle and superior temporal gyri [81]. Taken together, these findings provide some insights on how rs9329221 might confer antagonistic effects on SCZ and ASD.

Six of the implicated antagonistic SNPs were part of eQTLs that regulate gene expression in brain tissues, which suggests that brain structural changes may be driven by changes in gene expression [32]. Notably, the antagonistic SNPs influenced the expression of several genes that are known to be implicated in a wide range of neuronal processes like synaptic function, neuronal differentiation, or excitatory mechanisms, among others [82–85]. Among the strongest eQTL effects there was the T allele of rs3806843 (increased risk for SCZ and protective against MDD) that upregulates the expression of *PCDHA* genes which are suggested to be implicated in neuronal formation by establishing cell identity [86]. This observation is in line with the results of the conditional GWAS analysis for major neuropsychiatric disorders by Byrne et al. [20] that supported the notion that gene expression differences of *PCDHA* genes may contribute to antagonistic effects between SCZ and MDD.

We found that all eight implicated antagonistic SNPs were also associated with behavioral and cognitive traits that might be implicated in patients with neuropsychiatric disorders [87]. In particular, an association was identified between the C allele of rs2921036 (increased risk for SCZ and protective against ASD) and decreased measures of neuroticism [88]. Interestingly, rs2921036 is in moderate LD (*r*^*2*^ = 0.54 in CEU) with rs2945232 that was previously identified as a shared locus between neuroticism and SCZ [89]. The relevance of this genomic region for neuroticism measures in patients of ASD is less clear and future studies are warranted to explore to what extent the antagonistic effect might be shaped via behavioral or cognitive phenotypes [90].

In the exploratory voxel-wise whole-brain analysis in the FOR2107 study, we observed a significant negative association between the G allele dosage of rs301805 (increased risk for SCZ and protective against MDD) and GMV in the left superior temporal pole. We note that the labeled cluster (*k* = 998) extended to the left posterior orbital gyrus and the left insula, whereby both regions are known to be strongly interconnected to the temporal pole [91]. Interestingly, in our SNP-to-IDP analysis, we observed that the G allele of rs301805 was linked to a decrease of SA in the insula (Table 1). Both regions, the insula and the temporal pole, play an important role in emotional regulation and social cognition [91] and are implicated in SCZ [64, 75, 92]. In particular, patients with SCZ present age-related volume decline in the insula and temporal pole [64, 93]. Together, this might suggest a mediating role of brain structure for the effects of rs301805, whereby further molecular studies are warranted to follow-up this finding.

In addition, we found a significant positive association between the G allele dosage of rs1933802 (increased risk for SCZ and protective against MDD) and the left superior parietal region which is considered to play an important role in attention [94], internal body representation [95], as well as self-processing [96]. This link, however, has not been observed in our SNP-to-IDP analysis. Taken together, a VBM analysis of antagonistic SNPs including rs301805 and rs1933802 in larger cohorts (e.g., in the framework of the ENIGMA consortium) should be conducted to follow-up findings reported in the present study.

### Limitations and future directions

The present study had several limitations: First, we focused on the antagonistic SNPs identified by the currently largest cross-disorder GWAS meta-analysis of the PGC [24] wherein these SNPs have not been explored regarding their link to brain structure. Notably, other genetic studies used alternative methods to investigate genetic differences across neuropsychiatric disorders such as case-case GWAS which assess differences in allele frequencies across two disorders [21]. Moreover, the PGC-CDG2 GWAS meta-analysis [24] has reported that two antagonistic SNPs were associated with more than two disorders. For the other antagonistic SNPs, we are currently unable to rule out that these are associated with further neuropsychiatric disorders in addition to those two reported in the PGC-CDG2 GWAS meta-analysis [24]. Future studies should investigate through which mechanisms the genetic variants identified in other studies influence susceptibility to different neuropsychiatric disorders and examine which additional disorders might be associated with the antagonistic SNPs analyzed in the present study.

Second, within this study, single variant analyses were performed. Reasons for this were the limited number of antagonistic SNPs as well as their association with oppositely directed effects across various combinations of neuropsychiatric disorders. In addition, single variant analyses might be able to identify the underlying neurobiological pathways if the eleven antagonistic SNPs exert effects on different pathways that do not strongly overlap. Future cross-disorder GWAS might extend the set of antagonistic SNPs. Hence, with the availability of a greater number of SNPs with oppositely directed effects across two specific disorders, future studies should investigate the neurobiological correlates of their aggregated genetic scores.

Third, our findings of the SNP-to-IDP analysis mainly referred to the healthy population as these associations were reassessed from summary statistics of GWAS of brain structural phenotypes comprising predominantly healthy individuals. This approach benefited from a greater sample size and gave initial indications of the SNP effects on brain structure, but future case-control genomic imaging analyses are required to assess whether the SNP effects on brain structure are potentially more pronounced in patients with neuropsychiatric disorders.

Fourth, when interpreting the results of our SNP-to-IDP analysis and the voxel-wise whole brain analysis, it has to be considered that the data of the FOR2107 study were partly included in the GWAS of cortical phenotypes [35] and thus, that both analyses are not fully independent.

Lastly, we investigated the association between antagonistic SNPs and IDPs and thus, cannot make assumptions of causation. A true causal link between disorder risk and brain structure might exist if the antagonistic SNP affects biological pathways that influence brain circuitry whose disruption leads to a greater vulnerability for one neuropsychiatric disorder and a reduced vulnerability for the other disorder. However, the link between disorder risk and brain structure can also occur owing to variants in strong LD that act through independent pathways and are tagged by a third genetic variant [97]. Further fine-mapping and functional analyses of the eleven antagonistic SNPs are therefore warranted.

## Conclusion

The present study systematically investigated the influence of eleven antagonistic SNPs with opposite directional effects on neuropsychiatric disorders on brain structure. We showed that eight antagonistic SNPs were associated with brain structural phenotypes, especially with SA measurements, which were previously linked to neuropsychiatric disorders. These findings support our assumption that brain structural changes might contribute to the antagonistic effects of at least some of these SNPs. Furthermore, we found that specific antagonistic SNPs (i) were part of eQTLs which regulate gene expression in brain tissue, (ii) were associated with specific behavioral and cognitive traits, and (iii) showed significant associations with GMV in a voxel-wise whole-brain analysis (rs301805 and rs1933802). Our findings provide further insights how some antagonistic SNPs might modulate the risk of developing a specific neuropsychiatric disorder, thus advancing our understanding of the neurobiological mechanisms underlying these disorders.

## Supplementary information

Supplemental Material

## Data Availability

The associations of SNPs with image-derived phenotypes [35, 41] are available to all researchers via the ENIGMA-Vis tool (https://enigma-brain.org/enigmavis/). For the subcortical image-derived phenotypes [38], the GWAS summary statistics can be requested from the ENIGMA consortium (https://enigma.ini.usc.edu/research/download-enigma-gwas-results/). The statistics of the case-control MRI studies by the ENIGMA consortia can be accessed using the ENIGMA Toolbox (https://enigma-toolbox.readthedocs.io/en/latest/pages/04.loadsumstats/). The associations of SNPs with gene expression in brain tissues are publicly available in the GTEx (https://gtexportal.org/home/) and BRAINEAC (http://www.braineac.org/) databases. The associations of SNPs with cognitive-behavioral traits can be queried from the Open Targets Genetics portal (https://genetics.opentargets.org/). The data from the FOR2107 study is available from the corresponding authors upon reasonable request.
