# Supplementary material for: Associations between antagonistic SNPs for neuropsychiatric disorders and human brain structure
Source: Transl Psychiatry. 2024 Oct 2;14:406. doi: 10.1038/s41398-024-03098-1 (PMC11446931; doi:10.1038/s41398-024-03098-1)
Supplement: Supplementary file 1 — Supplemental Material [file 41398_2024_3098_MOESM1_ESM.pdf]

Supplementary Information

Supplementary Information 1
1. Bootstrapping Test 2
Methods 2
Results 3
2. FOR2107 Study 3
Sample Characteristics 3
Genotyping, Quality Control and Imputation 4
Acquisition and Preprocessing of Structural MRI Data 5
References 6
Supplementary Tables 12
Table S1 12
Table S2 13
Table S3 15
Table S4 18
Table S5 19
Table S6 22
Supplementary Figures 28
Figure S1 28
Figure S2 30

1. Bootstrapping Test

Methods

To test whether the observed number of significant SNP to IDP associations significantly differed for the eleven antagonistic SNPs, we performed a bootstrapping test. By this, we approximated a sampling distribution on the number of significant SNP to IDP associations across randomly sampled sets of eleven SNPs. We run separate comparisons based on resampling from two sets of SNPs, namely (i) the joined set of SNPs covered in all of the 78 summary statistics of each IDP (1–3) (*N*=6 559 812), and (ii) SNPs listed in the summary statistics of the PGC-CDG2 GWAS meta-analysis (4) (excluding subjects of 23andMe) with $p \leq 1.0 \times 10^{-06}$ that were covered in all of the 78 summary statistics (*N*=13 999).

For each set we randomly sampled *k* times eleven SNPs, (i) *k*=10 000 and (ii) *k*=1 000, without replacement and predefined seed. Next, we extracted the *p*-values of association for the eleven randomly drawn SNPs from the 78 summary statistics and corrected for multiple testing analog to our main analysis. To investigate whether the number of SNP to IDP associations found for the eleven antagonistic SNPs differs, we estimated the *p*-value along the bootstrapped distribution of the number of significant SNP to IDP associations by $p=\frac{1+\#\{t_k^* \geq t\}}{K+1}$, whereby #{} counts the occurrence of $t_k^* \geq t$ with t_k^* being the number of significant SNP to IDP associations for the *k*-th sampled set of eleven SNPs, and t being the number of significant SNP to IDP associations found for the antagonistic SNPs (5). Additionally, we obtained estimation of *p*-values for the number of significant SNP to IDP associations for SA, CT, and subcortical volume measures,

respectively. After correction for multiple testing using the Bonferroni method, significance was indicated by $p<6.25\times10^{-03}$ (eight tests).

Results

The number of significant SNP to IDP associations for the eleven antagonistic SNPs differed from the sampled distribution for both of the sets: Resampling from the joined set of SNPs across the 78 summary statistics showed significant differences for the total set of IDPs ($p=1.0\times10^{-04}$), the 35 SA ($p=1.0\times10^{-04}$), the 35 CT ($p=5.0\times10^{-03}$), and the eight subcortical volume measurements ($p=5.0\times10^{-04}$) (Supplementary Figure S1a-d). Similarly, resampling from the SNPs listed in the summary statistics of the PGC-CDG2 GWAS meta-analysis (4) with $p\leq1.0\times10^{-06}$ presented significant differences for the total set of IDPs ($p=3.0\times10^{-03}$), the 35 SA ($p=3.0\times10^{-03}$), and the eight subcortical volume measurements ($p=1.0\times10^{-03}$), as well as nominally significant associations with the 35 CT measurements ($p=2.0\times10^{-02}$) (Supplementary Figure S1e-h).

2. FOR2107 Study

Sample Characteristics

The FOR2107 study (6) is an ongoing longitudinal study designed to investigate the neurobiology of disorders across the affective disorders-psychosis spectrum. At time of analysis, the FOR2107 study comprised *N*=3 214 participants aged between 18 to 65 years that included healthy controls (HC) and patients in the affective disorders-psychosis spectrum diagnosed using the Diagnostic and Statistical Manual of Mental Disorders (DSM)-IV Structured Clinical Interview (7) including bipolar disorder (BIP), major depressive disorder (MDD), schizoaffective disorder (SZA), and schizophrenia (SCZ). At two sites, Marburg and Münster (Germany), multimodal data were

collected from each participant covering harmonized magnetic resonance imaging (MRI) scans, cognitive and psychological assessments, as well as biomaterial for generating genotyping data.

Genotyping, Quality Control and Imputation

The genotyping, quality control and imputation of the FOR2107 data set has been described in detail elsewhere (8). Briefly, the genotyping of the FOR2107 study was performed using the Infinium PsychArray-24 BeadChip (Illumina, San Diego, CA, US). Genetic quality control was conducted using PLINK v1.90 (9) and R v3.5.2.

For variant filtering, non-autosomal and ambiguous variants were dropped for further analyses. After alignment of alleles to the 1000 Genomes Phase 1 reference panel (10), variants not included in the panel were removed. Prior to the imputation variants with a call rate <98%, a MAF <1%, and/or a Hardy-Weinberg Equilibrium (HWE) test p-value $<1.0\times10^{-06}$ were excluded.

For sample filtering, samples with genotyping rates <98%, sex mismatches or other X-chromosomal linked conditions, genetic duplicates, cryptic relatedness (*pi-hat*≥12.5), and deviations of the autosomal or X-chromosomal heterozygosity rates (>4 standard deviations (SD) from the mean), and genetic ancestry components outlier (i.e. samples with >4 SD from the mean of the first eight multidimensional scaling ancestry components) were also removed. In total, n=2 241 participants remained for further analysis.

Pre-phasing was performed for each chromosome using SHAPEIT v2 (r837) (11). The imputation was performed using IMPUTE2 v2.3.2 (12,13) and the 1000 Genomes Phase 3 reference panel (10). Variants with a MAF of <1% and/or an INFO-score of <0.8 were removed. The genotype dosages of the eleven antagonistic SNPs were extracted from the imputed genetic

data of the FOR2107 study. Furthermore, based on the imputed SNP set multidimensional scaling (MDS) components were calculated using PLINK v1.9 (9). The first three MDS components were later included as covariates to adjust for population stratification.

Acquisition and Preprocessing of Structural MRI Data

T1-weighted anatomical 3D images were obtained in Marburg on a 3T Siemens Tim-Trio MR scanner with a 12-channel head matrix Rx-coil and in Münster on a 3T Siemens Prisma MR scanner with a 20-channel head matrix Rx-coil. For MRI acquisition the MP-RAGE sequence was used with following parameters: 176 sagittal slices in Marburg, 192 sagittal slices in Münster, field-of-view=256 mm and a final voxel resolution of 1×1×1 mm^3.

T1-weighted 3D images were preprocessed using the CAT-12 toolbox (14) version 1184 which builds on the SPM12 toolbox (15). The preprocessing was performed based on default parameters and included volumetric segmentation into gray matter, white matter, and cerebrospinal fluid. Using the volume-based diffeomorphic DARTEL algorithm (16) the gray matter volumes were reparametrized to MNI152 space for spatial normalization. Modulated gray matter volumes were smoothed using a Gaussian Kernel of 8 mm full width at half maximum. Finally, the total intracranial volume was extracted.

References

1. Grasby KL, Jahanshad N, Painter JN, Colodro-Conde L, Bralten J, Hibar DP, et al. The genetic architecture of the human cerebral cortex. *Science*. 2020; **367**.

2. Satizabal CL, Adams HHH, Hibar DP, White CC, Knol MJ, Stein JL, et al. Genetic architecture of subcortical brain structures in 38,851 individuals. *Nat Genet*. 2019; **51**:1624–36.

3. Hibar DP, Adams HHH, Jahanshad N, Chauhan G, Stein JL, Hofer E, et al. Novel genetic loci associated with hippocampal volume. *Nat Commun*. 2017; **8**.

4. Lee PH, Anttila V, Won H, Feng YCA, Rosenthal J, Zhu Z, et al. Genomic Relationships, Novel Loci, and Pleiotropic Mechanisms across Eight Psychiatric Disorders. *Cell*. 2019; **179**:1469-1482.e11.

5. Davison AC, Hinkley DV. Bootstrap methods and their application. Cambridge university press; 1997.

6. Kircher T, Wöhr M, Nenadic I, Schwarting R, Schratt G, Alferink J, et al. Neurobiology of the major psychoses: a translational perspective on brain structure and function—the FOR2107 consortium. *Eur Arch Psychiatry Clin Neurosci*. 2019; **269**:949–62.

7. Wittchen HU, Zaudig M, Fydrich T. SKID. Strukturiertes Klinisches Interview für DSM-IV. Achse I und II. Handanweisung. 1997;

8. Andlauer TFM, Mühleisen TW, Hoffstaedter F, Teumer A, Wittfeld K, Teuber A, et al. Genetic factors influencing a neurobiological substrate for psychiatric disorders. *Transl Psychiatry*. 2021; **11**:24.

9. Chang CC, Chow CC, Tellier LCAM, Vattikuti S, Purcell SM, Lee JJ. Second-generation PLINK: Rising to the challenge of larger and richer datasets. *Gigascience*. 2015; **4**.

10. Auton A, Abecasis GR, Altshuler DM, Durbin RM, Bentley DR, Chakravarti A, et al. A global reference for human genetic variation. *Nature*. 2015; **526**:68.

11. Delaneau O, Zagury JF, Marchini J. Improved whole-chromosome phasing for disease and population genetic studies. *Nat Methods*. 2013; **10**:5–6.

12. Howie BN, Donnelly P, Marchini J. A flexible and accurate genotype imputation method for the next generation of genome-wide association studies. *PLoS Genet*. 2009; **5**:e1000529.

13. Howie B, Fuchsberger C, Stephens M, Marchini J, Abecasis GR. Fast and accurate genotype imputation in genome-wide association studies through pre-phasing. *Nat Genet*. 2012; **44**:955–9.

14. Gaser C, Dahnke R, Thompson PM, Kurth F, Luders E, Alzheimer's Disease Neuroimaging Initiative. CAT – A Computational Anatomy Toolbox for the Analysis of Structural MRI Data. *bioRxiv*. 2022;1–38.

15. Penny WD, Friston KJ, Ashburner JT, Kiebel SJ, Nichols TE. Statistical parametric mapping: the analysis of functional brain images. Elsevier; 2011.

16. Ashburner J. A fast diffeomorphic image registration algorithm. *Neuroimage*. 2007; **38**:95–113.

17. Hoogman M, Bralten J, Hibar DP, Mennes M, Zwiers MP, Schweren LSJ, et al. Subcortical brain volume differences in participants with attention deficit hyperactivity

disorder in children and adults: a cross-sectional mega-analysis. *The Lancet Psychiatry*. 2017; **4**:310–9.

18. Hoogman M, Muetzel R, Guimaraes JP, Shumskaya E, Mennes M, Zwiers MP, et al. Brain imaging of the cortex in ADHD: A coordinated analysis of large-scale clinical and population-based samples. *Am J Psychiatry*. 2019; **176**:531–42.
19. Van Rooij D, Anagnostou E, Arango C, Auzias G, Behrmann M, Busatto GF, et al. Cortical and subcortical brain morphometry differences between patients with autism spectrum disorder and healthy individuals across the lifespan: Results from the ENIGMA ASD working group. *Am J Psychiatry*. 2018; **175**:359–69.
20. Hibar DP, Westlye LT, van Erp TGM, Rasmussen J, Leonardo CD, Faskowitz J, et al. Subcortical volumetric abnormalities in bipolar disorder. *Mol Psychiatry*. 2016; **21**:1710–6.
21. Hibar DP, Westlye LT, Doan NT, Jahanshad N, Cheung JW, Ching CRK, et al. Cortical abnormalities in bipolar disorder: An MRI analysis of 6503 individuals from the ENIGMA Bipolar Disorder Working Group. *Mol Psychiatry*. 2018; **23**:932–42.
22. Schmaal L, Veltman DJ, Van Erp TGM, Smann PG, Frodl T, Jahanshad N, et al. Subcortical brain alterations in major depressive disorder: findings from the ENIGMA Major Depressive Disorder working group. *Mol Psychiatry*. 2016; **21**:806–12.
23. Schmaal L, Hibar DP, Sämann PG, Hall GB, Baune BT, Jahanshad N, et al. Cortical abnormalities in adults and adolescents with major depression based on brain scans from 20 cohorts worldwide in the ENIGMA Major Depressive Disorder Working Group. *Mol*

Psychiatry. 2016; **22**:900–9.

24. Boedhoe PSW, Schmaal L, Abe Y, Alonso P, Ameis SH, Anticevic A, et al. Cortical abnormalities associated with pediatric and adult obsessive-compulsive disorder: Findings from the enigma obsessive-compulsive disorder working group. *Am J Psychiatry*. 2018; **175**:453–62.
25. Boedhoe PSW, Schmaal L, Abe Y, Ameis SH, Arnold PD, Batistuzzo MC, et al. Distinct subcortical volume alterations in pediatric and adult OCD: A worldwide meta- and mega-analysis. *Am J Psychiatry*. 2017; **174**:60–70.
26. Van Erp TGM, Hibar DP, Rasmussen JM, Glahn DC, Pearlson GD, Andreassen OA, et al. Subcortical brain volume abnormalities in 2028 individuals with schizophrenia and 2540 healthy controls via the ENIGMA consortium. *Mol Psychiatry*. 2015; **21**:547–53.
27. Van Erp TGM, Walton E, Hibar DP, Schmaal L, Jiang W, Glahn DC, et al. Cortical Brain Abnormalities in 4474 Individuals With Schizophrenia and 5098 Control Subjects via the Enhancing Neuro Imaging Genetics Through Meta Analysis (ENIGMA) Consortium. *Biol Psychiatry*. 2018; **84**:644–54.
28. Larivière S, Paquola C, Park B yong, Royer J, Wang Y, Benkarim O, et al. The ENIGMA Toolbox: multiscale neural contextualization of multisite neuroimaging datasets. *Nat Methods*. 2021; **18**:698–700.
29. Ramasamy A, Trabzuni D, Guelfi S, Varghese V, Smith C, Walker R, et al. Genetic variability in the regulation of gene expression in ten regions of the human brain. *Nat Neurosci*. 2014; **17**:1418–28.

30. Hibar DP, Westlye LT, Doan NT, Jahanshad N, Cheung JW, Ching CRK, et al. Cortical abnormalities in bipolar disorder: An MRI analysis of 6503 individuals from the ENIGMA Bipolar Disorder Working Group. *Mol Psychiatry*. 2018; **23**:932–42.

31. Ghoussaini M, Mountjoy E, Carmona M, Peat G, Schmidt EM, Hercules A, et al. Open Targets Genetics: systematic identification of trait-associated genes using large-scale genetics and functional genomics. *Nucleic Acids Res*. 2021; **49**:D1311–20.

32. Mountjoy E, Schmidt EM, Carmona M, Schwartzentruber J, Peat G, Miranda A, et al. An open approach to systematically prioritize causal variants and genes at all published human GWAS trait-associated loci. *Nat Genet*. 2021; **53**:1527–33.

33. Buniello A, Macarthur JAL, Cerezo M, Harris LW, Hayhurst J, Malangone C, et al. The NHGRI-EBI GWAS Catalog of published genome-wide association studies, targeted arrays and summary statistics 2019. *Nucleic Acids Res*. 2019; **47**:D1005–12.

34. Eysenck SBG, Eysenck HJ, Barrett P. A revised version of the psychoticism scale. *Pers Individ Dif*. 1985; **6**:21–9.

35. Smith DJ, Escott-Price V, Davies G, Bailey MES, Colodro-Conde L, Ward J, et al. Genome-wide analysis of over 106 000 individuals identifies 9 neuroticism-associated loci. *Mol Psychiatry*. 2016; **21**:749.

36. Tzourio-Mazoyer N, Landeau B, Papathanassiou D, Crivello F, Etard O, Delcroix N, et al. Automated Anatomical Labeling of Activations in SPM Using a Macroscopic Anatomical Parcellation of the MNI MRI Single-Subject Brain. *Neuroimage*. 2002; **15**:273–89.

37. Rolls ET, Huang CC, Lin CP, Feng J, Joliot M. Automated anatomical labelling atlas 3.

Neuroimage. 2020; **206**:116189.

38. Mordkoff JT. A Simple Method for Removing Bias From a Popular Measure of Standardized Effect Size: Adjusted Partial Eta Squared. *Adv Methods Pract Psychol Sci*. 2019; **2**:228–32.

39. Amunts K, Mohlberg H, Bludau S, Zilles K. Julich-Brain: A 3D probabilistic atlas of the human brain's cytoarchitecture. *Science*. 2020; **369**:988–92.

Supplementary Tables

Table S1

Overview of GWAS of Brain Structural Phenotypes by the ENIGMA and CHARGE Consortia

Study	Brain measures	Brain structural phenotype	Sample size (discovery cohort)
Grasby et al. (2020) (1)	CT, SA	Average CT, total SA, 34 CT and 34 SA measurements of the following Desikan-Killiany regions: Frontal pole, medial orbitofrontal, lateral orbitofrontal, rostral anterior cingulate, caudal anterior cingulate, superior frontal, rostral middle frontal, pars orbitalis, pars triangularis, pars opercularis, caudal middle frontal, paracentral, precentral, postcentral, precuneus, superior parietal, inferior parietal, posterior cingulate, isthmus cingulate, insula, supramarginal, entorhinal, parahippocampal, fusiform, temporal pole, inferior temporal, middle temporal, superior temporal, banks of the superior temporal sulcus, transverse temporal, lingual, pericalcarine, cuneus, and lateral occipital	33 281
Hibar et al. (2017) (3)	Volume	Hippocampal volume	26 814
Satizabal et al. (2019) (2)	Volume	Seven volume measurements of the accumbens, amygdala, brainstem, caudate nucleus, globus pallidus, putamen, and thalamus	37 741

Sample size was taken from the requested GWAS summary statistics. CT, cortical thickness; SA, surface area.

Table S2

Overview of Case-control MRI Studies by the ENIGMA Consortium

Study	Disorder	Brain measures	Cases/controls	Covariates	Multiple testing correction
Hoogman et al. (2017) (17)	ADHD	Volume	1713/1529	age, sex, ICV, scanner site	FDR at q=0.05
Hoogman et al. (2019) (18)	ADHD	CT, SA	2246/1934	age, sex, ICV[1]	FDR at q=0.05
van Rooij et al. (2018) (19)	ASD	CT, SA, Volume	1658/1606	age, sex, IQ, ICV[2]	FDR[3]
Hibar et al. (2016) (20)	BIP	Volume	1710/2594	age, sex, ICV	$p<4.91\times10^{-03}$ for FDR at q=0.05
Hibar et al. (2018) (21)	BIP	CT, SA	1837/2582	age, sex, ICV[1]	FDR at q=0.05
Schmaal et al. (2016) (22)	MDD	Volume	1728/7199	age, sex, ICV, scanner site	Bonferroni correction $p<5.6\times10^{-03}$
Schmaal et al. (2017) (23)	MDD	CT, SA	2148/7957	age, sex, scanner site	FDR at q=0.05
Boedhoe et al. (2018) (24)	OCD	Volume	1830/1759	age, sex, ICV, scanner site	Bonferroni correction $p<5.6\times10^{-03}$
Boedhoe et al. (2017) (25)	OCD	CT, SA	1905/1760	age, sex, ICV[1], scanner site	FDR at q=0.05
van Erp et al. (2016) (26)	SCZ	Volume	2028/2540	age, sex, ICV, scanner site	Bonferroni correction $p<5.6\times10^{-03}$
van Erp et al. (2018) (27)	SCZ	CT, SA	4474/5098	age, sex	FDR[3]

We note that covariates and multiple testing correction were retrieved from the summary statistics overview by the ENIGMA toolbox (28) (*https://enigma-toolbox.readthedocs.io/en/latest/pages/04.loadsumstats/index.html*) and the manuscripts. [1]only for SA measures. [2]only for subcortical volume measures. [3]q was not further specified in the manuscript. ADHD, attention deficit hyperactivity disorder; ASD, autism spectrum disorder; BIP, bipolar disorder; CT, cortical thickness; FDR, false discovery rate; ICV, intracranial volume; MDD, major depressive disorder; OCD, obsessive-compulsive disorder; SA, surface area; SCZ, schizophrenia.

Table S3

Antagonistic SNPs and eQTLs within Brain Tissues as Registered in GTEx and BRAINEAC

rsID	Gene(s)	GTEx	BRAINEAC			
			AveALL	FCTX	OCTX	TCTX
rs2388334	n.s.					
rs301805	*RERE*	CAU: $p=3.0\times10^{-06}$; HIPP: $p=4.0\times10^{-06}$; NAcc: $p=1.7\times10^{-05}$; CTX: $p=4.8\times10^{-05}$	6.3×10^{-08}	6.7×10^{-05}		
	GPR157			3.1×10^{-04}		
rs75595651[1]	n.s.					
rs1933802	*LIN28B-AS1*	CAU: $p=2.3\times10^{-06}$; PUT: $p=4.8\times10^{-07}$				
	HACE1	CTX: $p=6.1\times10^{-06}$				
rs6748341	*CUL3*	CTX: $p=4.9\times10^{-06}$				
rs3806843	*PCDHA*[2]	CAU: $p=8.1\times10^{-10}$; NAcc: $p=2.2\times10^{-09}$; CBh: $p=2.9\times10^{-14}$; CTX: $p=2.2\times10^{-12}$; PUT: $p=2.0\times10^{-06}$; CB: $p=4.0\times10^{-15}$; FCTX (BA9): $p=3.0\times10^{-07}$; ACC (BA24): $p=1.5\times10^{-06}$; HYPO: $p=2.1\times10^{-06}$	5.6×10^{-08}			2.8×10^{-05}
	PCDHB[2]		2.2×10^{-04}			
	PCDHG[2]		3.2×10^{-04}		2.4×10^{-04}	
	HARS	CBh: $p=3.3\times10^{-05}$; CTX: $p=5.9\times10^{-05}$; CB: $p=1.9\times10^{-06}$				
	SRA1	HIPP: $p=1.2\times10^{-05}$	4.0×10^{-10}	1.9×10^{-07}		
	WDR55	CAU: $p=5.0\times10^{-05}$; CTX: $p=1.2\times10^{-05}$; CB: $p=1.8\times10^{-07}$				

Continues on the next page

Table S3 continued

rsID	Gene(s)	GTEx	BRAINEAC			
			AveALL	FCTX	OCTX	TCTX
rs3806843	*ZMAT2*	CAU: $p=3.2\times10^{-06}$; NAcc: $p=3.6\times10^{-05}$; CBh: $p=9.1\times10^{-06}$; CTX: $p=3.4\times10^{-06}$; PUT: $p=1.3\times10^{-05}$; CB: $p=1.1\times10^{-06}$				
	TMCO6	CBh: $p=5.0\times10^{-10}$; CB: $p=4.3\times10^{-09}$				
rs9329221	*LINCR-0001*	CAU: $p=3.2\times10^{-08}$; HIPP: $p=2.1\times10^{-05}$				
rs2921036	*ERI1*		1.6×10^{-09}		1.3×10^{-05}	
	FAM85B	CAU: $p=8.1\times10^{-10}$; HIPP: $p=7.5\times10^{-12}$; NAcc: $p=3.6\times10^{-14}$; CBh: $p=4.4\times10^{-11}$; CTX: $p=3.0\times10^{-16}$; PUT: $p=1.5\times10^{-07}$; CB: $p=1.4\times10^{-15}$; FCTX (BA9): $p=1.4\times10^{-13}$; ACC (BA24): $p=1.8\times10^{-08}$; HYPO: $p=2.6\times10^{-13}$; AMY: $p=3.0\times10^{-12}$; STNG: $p=2.3\times10^{-07}$				

Six of the eight antagonistic SNPs that were significantly associated with at least one brain image-derived phenotype were part of an eQTL in a specific brain tissue. Results are reported with $p<4.0\times10^{-04}$ corresponding to a Bonferroni correction for eight SNPs and 16 brain tissues. Note that pseudogenes were excluded. [1]rs75595651 was replaced by the proxy SNP rs77087420. [2]marks gene clusters, whereby we report the *p*-value with the lowest value among the genes of that cluster. ACC, anterior cingulate cortex; AMY, amygdala; AveALL, average across all ten brain tissues obtained in the database of BRAINEAC (29); BA9, Brodmann Area 9; BA24, Brodmann Area 24; BRAINEAC, Brain eQTL Almanac; CAU, caudate; CB, cerebellum; CBh, cerebellar hemisphere; CTX, cortex; FCTX, frontal

cortex; GTEx, Genotype-Tissue Expression database, HIPP, hippocampus; HYPO, hypothalamus; NAcc, nucleus accumbens; n.s., not significant; OCTX, occipital cortex; PUT, putamen; STNG, substantia nigra; TCTX, temporal cortex.

Table S4

Significant Brain Structural Alterations in Patients with Neuropsychiatric Disorders

Disorder	Measure	Brain region	d_{left}	$p_{FDR,\ left}$	d_{right}	$p_{FDR,\ right}$
BIP (30)	CT	caudal ant. cingulate	-0.095	4.2×10^{-02}	n.s.	n.s.
		rostral ant. cingulate	-0.153	3.8×10^{-05}	n.s.	n.s.
MDD (23)	CT	post. cingulate	-0.099	1.8×10^{-02}	-0.093	2.2×10^{-02}
		rostral ant. cingulate	-0.130	3.0×10^{-02}	-0.098	3.4×10^{-02}
SCZ (26,27)	CT[1]	post. cingulate	-0.298	4.7×10^{-21}	-0.310	1.2×10^{-26}
		supramarginal	-0.395	4.9×10^{-15}	-0.386	1.3×10^{-17}
	SA[2]	caudal ant. cingulate	-0.128	5.1×10^{-04}	-0.156	1.2×10^{-08}
		post. cingulate	-0.117	1.5×10^{-03}	-0.125	1.3×10^{-03}
		insula	-0.122	3.5×10^{-03}	-0.113	4.3×10^{-03}
		lateral orbitofrontal	-0.179	4.2×10^{-05}	-0.150	1.1×10^{-04}
		lingual	-0.148	7.8×10^{-05}	-0.168	8.3×10^{-07}
		pars opercularis	-0.151	9.0×10^{-06}	-0.146	2.0×10^{-07}
		pericalcarine	-0.133	1.7×10^{-03}	-0.107	3.8×10^{-03}
		superior temporal	-0.196	9.2×10^{-09}	-0.195	9.3×10^{-07}
		transverse temporal	-0.151	7.4×10^{-03}	-0.169	9.0×10^{-09}
**Disorder**	**Measure**	**Brain region**	d		p	
SCZ	Vol.	nucleus accumbens	-0.25		1.5×10^{-05}	

Table S4 shows case-control differences denoted by Cohen's d that were observed as significant after multiple testing correction (cortical IDPs: $p_{FDR}<0.05$; subcortical IDPs in SCZ: $p<5.6\times10^{-03}$) in the respective MRI study by the ENIGMA Consortium. Case-control differences were significant for patients of BIP, MDD, and SCZ only. Note that our analysis only comprised IDPs that were significantly associated with an antagonistic SNP. For cortical IDPs in BIP and MDD as well as subcortical IDPs in SCZ statistics were taken from Table 1 in (23,26,30). [1]Statistics were taken from the Supplementary Table 4a, and [2]Table 5a in (27). BIP, bipolar disorder; CT, cortical thickness; FDR, false discovery rate; MDD, major depressive disorder; n.s., not significant; SA, surface area; SCZ, schizophrenia.

Table S5

Trait Associations of the Antagonistic SNPs Listed in Open Targets Genetics

<table>
<tr><th>rsID</th><th>Category</th><th>EA</th><th>Associated traits [p-value; β; Study accession number]</th></tr>
<tr><td>rs2388334</td><td>Behavior</td><td>G</td><td>Time spend outdoors in summer [$p=6.9\times10^{-19}$; $\beta=-0.02$; NEALE2_1050]; Average total household income before tax [$p=1.8\times10^{-18}$; $\beta=0.02$; NEALE2_738]; Job involves mainly walking or standing [$p=3.0\times10^{-17}$; $\beta=-0.03$; NEALE2_806]; Job involves heavy manual or physical work [$p=4.0\times10^{-16}$; $\beta=-0.02$; NEALE2_816]; Time spent using computer [$p=3.5\times10^{-13}$; $\beta=0.02$; NEALE2_1080]; Participation in an health questionnaire (not invited vs invited) [$p=3.3\times10^{-11}$; $\beta=-0.006$; GCST90012794]; Time spent watching television (tv) [$p=1.3\times10^{-10}$; $\beta=-0.01$; NEALE2_1070]; Time spent outdoors in winter [$p=1.6\times10^{-10}$; $\beta=-0.01$; NEALE2_1060]; Number of days/week walked 10+ minutes [$p=2.5\times10^{-09}$; $\beta=-0.03$; NEALE2_864]</td></tr>
<tr><td></td><td>Cognition</td><td></td><td>Intelligence [$p=3.6\times10^{-29}$; $\beta=0.03$; GCST006250]; Cognitive performance [$p=1.7\times10^{-26}$; $\beta=0.03$; GCST006572]; Fluid intelligence score [$p=4.8\times10^{-11}$; $\beta=0.06$; NEALE2_20016_raw]</td></tr>
<tr><td></td><td>Education</td><td></td><td>College or university degree | qualifications [$p=2.8\times10^{-37}$; $\beta=0.06$; NEALE2_6138_1]; A levels/as levels or equivalent | qualifications [$p=7.0\times10^{-14}$; $\beta=0.04$; NEALE2_6138_2]; Cses or equivalent | qualifications [$p=9.2\times10^{-10}$; $\beta=-0.04$; NEALE2_6138_4]; Educational attainment [$p=3.2\times10^{-09}$; $\beta=0.03$; GCST003496]; Age completed full time education [$p=5.4\times10^{-09}$; $\beta=0.01$; NEALE2_845]; Year ended full time education [$p=2.4\times10^{-08}$; $\beta=0.09$; NEALE2_22501_raw]</td></tr>
<tr><td></td><td>Food pref.</td><td></td><td>Muesli | cereal type [$p=3.1\times10^{-13}$; $\beta=0.05$; NEALE2_1468_4]; Wholemeal or wholegrain | bread type [$p=2.1\times10^{-10}$; $\beta=0.03$; NEALE2_1448_3]; Hot drink temperature [$p=7.9\times10^{-10}$; $\beta=-0.008$; NEALE2_1518]; Cereal intake [$p=2.3\times10^{-09}$; $\beta=0.01$; NEALE2_1458]; White | bread type [$p=4.4\times10^{-09}$; $\beta=-0.03$; NEALE2_1448_1]</td></tr>
</table>

Continues on the next page

Table S5 continued

rsID	Category	EA	Associated traits [*p*-value; *β*; Study accession number]
rs301805	Neuroticism	G	Feeling tense [p=7.6×10^{-09}; β=-0.01; GCST006952]; Feeling miserable [p=2.7×10^{-08}; β=-0.01; GCST006943]; Tense / 'highly strung' [p=3.9×10^{-08}; β=-0.04; NEALE2_1990]; Depressed affect [p=4.2×10^{-08}; β=-0.01; GCST006475]
	Chronotype		Daytime nap [p=6.9×10^{-09}; β=0.007; GCST011494]
rs75595651	Neuroticism	T	Fed-up feelings [p=6.2×10^{-10}; β=-0.06; NEALE2_1960; p=3.0×10^{-08}; β=-0.03; GCST006947]; Miserableness [p=1.1×10^{-09}; β=-0.06; NEALE2_1930; p=1.5×10^{-08}; β=-0.03; GCST006943]
rs1933802	Neuroticism	G	Feeling guilty [p=7.3×10^{-09}; β=0.01; GCST006945]
rs6748341	Behavior	G	Age at first sexual intercourse [p=1.1×10^{-11}; β=0.01]; Walk | types of transport used (excluding work) [p=4.3×10^{-08}; β=0.03; NEALE2_6162_2]
rs3806843	Cognition	C	Intelligence [p=1.4×10^{-08}; β=0.02; GCST006250]
rs9329221	Behavior	T	Age first had sexual intercourse [p=1.0×10^{-14}; β=-0.07; NEALE2_2139_raw; p=4.2×10^{-13}; β=-0.02; GCST90000047]
	Neuroticism		Neuroticism [p=8.0×10^{-21}; β=-0.05; GCST005232; p=1.7×10^{-18}; β=-0.07; NEALE2_20127_raw; p=1.6×10^{-15}; β=-0.02; GCST005327; p=6.6×10^{-15}; β=-0.03; GCST003770]; Worrier / anxious feelings [p=3.4×10^{-18}; β=-0.04; NEALE2_1980]; Irritability [p=2.3×10^{-14}; β=-0.04; NEALE2_1940]; Miserableness [p=2.2×10^{-13}; β=-0.03; NEALE2_1930]; Nervous feelings [p=1.2×10^{-12}; β=-0.04; NEALE2_1970]
	Chronotype		Sleep duration [p=2.3×10^{-08}; β=0.01; NEALE2_1160]
	Food pref.		Cheese intake [p=5.1×10^{-13}; β=-0.02; NEALE2_1408]
rs2921036	Behavior	C	Age first had sexual intercourse [p=6.4×10^{-13}; β=-0.07; NEALE2_2139_raw; p=7.1×10^{-12}; β=-0.01; GCST90000047]

Continues on the next page

Table S5 continued

rsID	Category	EA	Associated traits [p-value; β; Study accession number]
rs2921036	Neuroticism	C	Neuroticism score [p=6.2×10^{-26}; β=-0.09; NEALE2_20127_raw; p=8.0×10^{-26}; β=-0.06; GCST005232; p=8.3×10^{-16}; β=-0.02; GCST005327; p=1.2×10^{-14}; β=-0.03; GCST003770]; Worrier / anxious feelings [p=9.5×10^{-23}; β=-0.05; NEALE2_1980]; Irritability [p=3.0×10^{-15}; β=-0.04; NEALE2_1940]; Nervous feelings [p=4.2×10^{-15}; β=-0.04; NEALE2_1970]; Miserableness [p=2.0×10^{-13}; β=-0.03; NEALE2_1930]; Worry too long after embarrassment [p=1.2×10^{-12}; β=-0.03; NEALE2_2000]; Fed-up feelings [p=2.2×10^{-12}; β=-0.03; NEALE2_1960]; Tense / 'highly strung' [p=3.3×10^{-12}; β=-0.04; NEALE2_1990]; Sensitivity / hurt feelings [p=1.9×10^{-10}; β=-0.03; NEALE2_1950]

Associated traits with p<5×10^{-08} were reported with the respective study accession number from Open Targets Genetics (31,32). Here numbers starting with 'GCST' refer to studies retrieved from the NHGRI-EBI GWAS Catalog (33) and those with 'NEALE2' refer to GWAS analyses using the UKBB data (*http://www.nealelab.is/uk-biobank*). β denotes the effect size in relation to the effect allele. Note that we assigned traits to the category 'Neuroticism' based on the items of the neuroticism scale of Eysenck Personality Questionnaire-Revised Short Form (34) that were part of the mental health factors assessed in the UKBB (35). EA, effect allele; GWAS, genome-wide association study; pref., preferences; SNP, single-nucleotide polymorphism.

Table S6

Whole Brain Analysis for the Eleven Antagonistic SNPs in the FOR2107 Study

rsID	EA/ OA	Risk	Prot.	Dir.	Cluster labelling	Cluster size	MNI152 space (peak voxel)			*T*-value	p_{FWE}-value	η^2
						(*k*)	*x*	*y*	*z*			
rs2388334	G/A	ASD BIP	TS	Pos.	Angular_L	148	-57	-58	32	3.95	0.331	0.010
					Temporal_Sup_L	49	-52	-44	14	3.55	0.763	0.008
					Outside	15	34	-3	-26	3.47	0.844	0.008
					Occipital_Mid_L	17	-44	-88	2	3.33	0.938	0.007
					Temporal_Sup_R	42	66	-30	2	3.31	0.945	0.007
					Temporal_Mid_L	14	-62	-60	18	3.29	0.955	0.007
				Neg.	Cerebellum_3_R	39	10	-36	-18	3.34	0.946	0.007
					Frontal_Mid_2_L	18	-28	28	44	3.31	0.974	0.007
					Cerebellum_3_L	26	-9	-36	-15	3.23	0.964	0.007
rs301805	G/T	SCZ	MDD	Pos.	Precuneus_R	18	10	-62	44	3.45	0.873	0.007
				Neg.	**Temporal_Pole_Sup_L**	**998**	**-28**	**10**	**-22**	**4.85**	**0.012**	**0.015**
					OFCpost_R	274	26	12	-22	3.78	0.526	0.009
					Lingual_L	81	-14	-40	-2	3.60	0.734	0.008
					Outside	34	14	-16	-21	3.58	0.752	0.008
					Temporal_Inf_R	208	52	-4	-34	3.57	0.767	0.008
					Hippocampus_R	55	26	-9	-20	3.22	0.981	0.006
					ParaHippocampal_R	25	32	-1	-22	3.19	0.985	0.006
					Lingual_R	15	15	-42	-6	3.19	0.985	0.006

Continues on the next page

Table S6 continued

rsID	EA/ OA	Risk	Prot.	Dir.	Cluster labelling	Cluster size (*k*)	MNI152 space (peak voxel) *x*	*y*	*z*	*T*-value	p_{FWE}-value	η^2
rs301805	G/T	SCZ	MDD	Neg.	ParaHippocampal_L	10	-15	-16	-22	3.18	0.986	0.006
rs75595651	T/C	BIP	MDD	Pos.	Calcarine_L	490	-2	-100	-6	3.81	0.470	0.009
					Fusiform_L	35	-39	-60	-16	3.54	0.776	0.008
					Frontal_Sup_2_R	26	21	52	39	3.32	0.973	0.007
				Neg.	Outside	16	15	-15	-32	3.36	0.920	0.007
rs1933802	G/C	MDD	SCZ	Pos.	**Parietal_Sup_L**	**448**	**-20**	**-69**	**62**	**4.62**	**0.029**	**0.013**
					Precuneus_R	957	8	-46	54	4.28	0.108	0.011
					Lingual_L	387	-9	-68	-4	4.16	0.166	0.011
					Precuneus_L	79	-16	-42	58	3.81	0.461	0.009
					Frontal_Mid_2_L	20	-34	45	-6	3.76	0.514	0.009
					Frontal_Med_Orb_L	199	-2	69	-2	3.74	0.537	0.009
					Calcarine_L	559	-3	-102	-2	3.69	0.597	0.009
					Frontal_Sup_2_R	143	20	63	-4	3.40	0.885	0.007
					Parietal_Sup_R	23	30	-58	62	3.38	0.902	0.007
					Outside	66	-15	46	-10	3.37	0.906	0.007
					Rolandic_Oper_R	23	52	2	8	3.24	0.967	0.007
					Outside	14	-28	-98	-15	3.23	0.968	0.007
				Neg.	Cerebellum_7b_R	37	46	-52	-54	3.50	0.803	0.008
					Parietal_Inf_L	18	-44	-56	56	3.29	0.948	0.007
					Outside	19	0	-10	2	3.22	0.971	0.006

Continues on the next page

Table S6 continued

rsID	EA/ OA	Risk	Prot.	Dir.	Cluster labelling	Cluster size (k)	MNI152 space (peak voxel) x	y	z	T-value	p_{FWE}-value	η^2
rs6748341	G/C	ANO	SCZ	Pos.	Fusiform_L	183	-28	-28	-22	3.83	0.421	0.009
					Temporal_Sup_R	56	64	-21	15	3.65	0.626	0.008
					Hippocampus_R	42	39	-33	-9	3.53	0.756	0.008
					Temporal_Sup_R	10	54	-20	9	3.21	0.989	0.006
				Neg.	ACC_pre_R	19	14	46	21	3.50	0.790	0.008
rs3806843	C/T	MDD	SCZ	Pos.	Outside	29	32	-9	14	3.42	0.871	0.008
				Neg.	Outside	302	6	-74	-46	3.57	0.733	0.008
					Outside	44	18	-93	-21	3.51	0.793	0.008
					Temporal_Inf_R	55	46	-16	-40	3.45	0.847	0.008
					Cerebellum_6_R	28	12	-69	-27	3.32	0.931	0.007
rs9329221	T/G	SCZ	ASD	Pos.	Temporal_Mid_R	95	39	-64	18	3.77	0.510	0.009
					Temporal_Mid_L	39	-69	-26	-18	3.52	0.788	0.008
					Temporal_Mid_L	113	-58	-66	-2	3.47	0.838	0.008
					Cingulate_Mid_R	37	8	-9	39	3.36	0.916	0.007
					Hippocampus_L	74	-28	-27	-10	3.34	0.925	0.007
					Hippocampus_R	59	32	-28	-8	3.26	0.961	0.007
				Neg.	Outside	57	18	-21	-33	4.23	0.129	0.011
					Occipital_Mid_R	508	30	-84	21	4.08	0.218	0.011
					Lingual_L	19	-20	-68	-2	3.58	0.723	0.008
					Cerebellum_4_5_L	262	-6	-57	-20	3.38	0.906	0.007

Continues on the next page

Table S6 continued

rsID	EA/ OA	Risk	Prot.	Dir.	Cluster labelling	Cluster size (*k*)	MNI152 space (peak voxel) *x*	*y*	*z*	*T*-value	p_{FWE}-value	η^2
rs2921036	C/T	SCZ	ASD	Pos.	Postcentral_L	564	-33	-45	52	4.27	0.114	0.012
					Cingulate_Mid_R	619	8	-9	38	4.09	0.207	0.011
					Outside	219	9	-39	28	3.92	0.347	0.010
					Precuneus_L	47	-4	-46	75	3.63	0.662	0.008
					Hippocampus_L	79	-28	-28	-8	3.58	0.716	0.008
				Neg.	Cerebellum_6_L	1204	-3	-66	-16	4.41	0.066	0.012
					Occipital_Sup_R	321	28	-86	24	3.68	0.603	0.009
					Cerebellum_6_L	56	-22	-64	-14	3.34	0.924	0.007
rs2867673	C/T	SCZ	ASD	Pos.	Parietal_Sup_R	19	21	-74	54	3.46	0.859	0.007
				Neg.	Rolandic_Oper_L	417	-48	2	10	4.11	0.206	0.010
					Outside	224	20	12	-42	3.85	0.441	0.009
					Lingual_R	13	9	-40	-4	3.26	0.966	0.007
					OFCpost_L	10	-21	12	-18	3.18	0.986	0.006
rs9511168	A/C	ADHD	ANO	Pos.	Precuneus_R	97	18	-54	26	3.66	0.641	0.008
					Frontal_Sup_2_L	37	-16	60	30	3.56	0.752	0.008
					Cuneus_R	44	10	-76	28	3.54	0.772	0.008
					Fusiform_L	176	-34	0	-34	3.48	0.833	0.008
					Olfactory_R	60	10	26	-12	3.39	0.897	0.007
					Paracentral_Lobule_L	18	-2	-20	76	3.33	0.933	0.007
				Neg.	OFCmed_R	67	21	48	-21	3.50	0.806	0.008

Continues on the next page

Table S6 continued

rsID	EA/ OA	Risk	Prot.	Dir.	Cluster labelling	Cluster size (k)	MNI152 space (peak voxel) x	y	z	T-value	p_{FWE}-value	η^2
rs9511168	A/C	ADHD	ANO	Neg	Precuneus_L	16	-9	-45	54	3.37	0.911	0.007
rs1363105	T/C	ANO	ADHD	Pos.	Frontal_Inf_Tri_R	565	51	24	18	4.13	0.190	0.011
			ASD		Outside	96	16	-21	-30	3.89	0.391	0.009
			MDD		Calcarine_L	172	-8	-99	-6	3.74	0.555	0.009
					Lingual_R	71	10	-56	2	3.51	0.815	0.008
					Calcarine_R	42	15	-69	4	3.43	0.884	0.007
					Temporal_Inf_R	24	50	-60	-4	3.33	0.941	0.007
					Frontal_Sup_2_R	21	22	12	58	3.25	0.970	0.007
				Neg.	Frontal_Mid_2_R	13	34	9	38	3.30	0.954	0.007
					Frontal_Mid_2_L	28	-36	14	38	3.29	0.956	0.007

Whole-brain analyses were conducted using a multiple regression design in the CAT12 toolbox (14). Positive and negative associations between gray matter volume and the genotype dosages were reported for clusters with size k>10 and $p_{uncorrected}<1\times10^{-3}$. Significant associations with $p_{FWE}<0.05$ (peak-level Family Wise Error (FWE) correction for multiple comparison) were marked as bold. Results of the two SNPs, rs301805 and rs1933802, that show an association with GMV at $p_{FWE}<0.05$, are visualized in Figure 4. We used the automated anatomical labelling atlas version 3 (36,37) to annotate the clusters anatomically and presented the label with the highest cluster proportion. The partial effect size $\eta^2$was computed based on T-values and its degree of freedom (Equation 4 in (38)). ACC_pre, anterior cingulate cortex pregenual; Dir., direction of association; EA, effect allele; Inf, inferior; L, left; Med, medial; Mid, middle; OA,

other allele; OFCmed, medial orbital gyrus; OFCpost, posterior orbital gyrus; Oper, operculum; Orb, orbitalis; Prot., protective; R, right; ROI, region of interest; Sup, superior; Tri, triangular.

Supplementary Figures

Figure S1

Results of the Bootstrapping Test

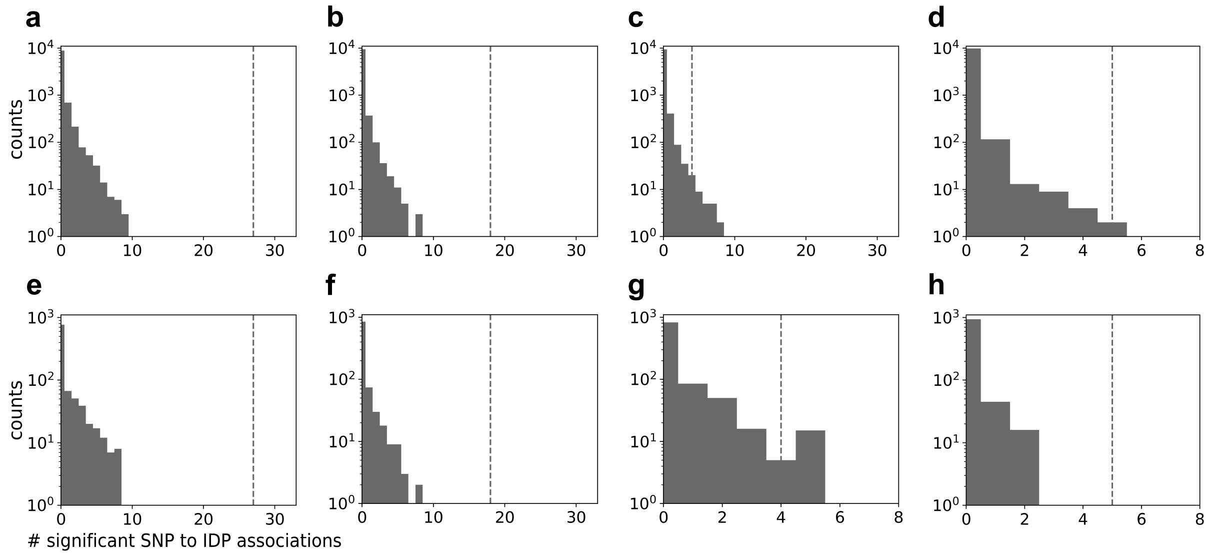

The number of significant SNP to IDP associations for the eleven antagonistic SNPs was compared to the sampled distribution of the number of significant SNP to IDP associations obtained by resampling sets of eleven SNPs. Figure S1 shows the approximated distribution of the number of significant SNP to IDP associations obtained by resampling eleven SNPs from the following SNP sets: **a-d** SNPs randomly drawn from the joined set of SNPs across the 78 summary statistics of each IDP (1–3); **e-h** SNPs randomly drawn from SNPs identified in the PGC-CDG2 GWAS meta-analysis (4) with $p \leq 1 \times 10^{-06}$ and covered in the joined set of SNPs across the summary statistics of 78 IDPs. Note that the number of significant SNP to IDP associations is shown across all 78 IDPs (**a**,**e**), 35 IDPs for surface area (**b**,**f**), 35 IDPs for cortical thickness (**c**,**g**), and eight IDPs for subcortical volume measurements (**d**,**h**). Horizontal lines indicate the number of significant SNP

to IDP associations observed for the eleven antagonistic SNPs. IDP, image-derived phenotype; SNP, single-nucleotide polymorphism.

Figure S2

Visualization of Significant Clusters (p_{FWE}<0.05) and their Peak Voxel from the Whole-Brain Analysis in the FOR2107 Study

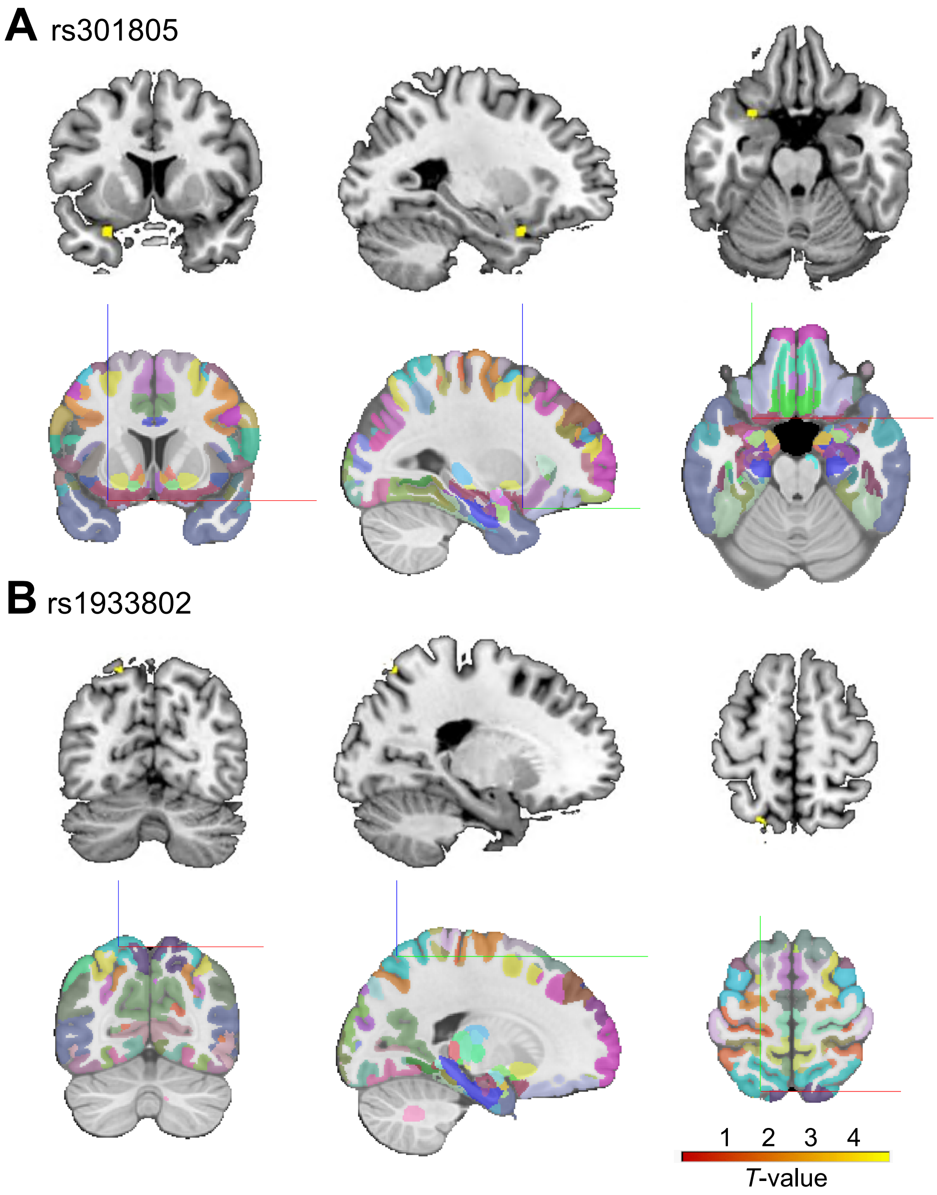

A The G allele of rs301805 was significantly negatively associated (p_{FWE}<0.05) with a GMV cluster that was labeled as left superior temporal pole by the anatomical labelling atlas v3 (36,37).

The corresponding peak-voxel *x*/*y*/*z*=-28/10/-22 was mapped to the left Frontal-to-Temporal-II GapMap based on the Julich Brain Atlas v3.1 (39) and is depicted in the bottom row using the EBRAINS viewer (*https://atlases.ebrains.eu/viewer/#/).* **B** The G allele of rs1933802 was significantly positively associated ($p_{FWE}<0.05$) with a GMV cluster that was labeled as left superior parietal region by the anatomical labelling atlas v3 (36,37). Similarly to **A**, the corresponding peak voxel (*x*/*y*/*z*=-20/-69/62) is depicted in the bottom row using the EBRAINS viewer and was mapped to the left Area 7A of the superior parietal lobe based on the Julich Brain Atlas v3.1 (39). FWE, family-wise error; GMV, gray matter volumes.
